# Insights into optimization of oleaginous fungi – genome-scale metabolic reconstruction and analysis of *Umbelopsis* sp. WA50703

**DOI:** 10.1016/j.csbj.2025.03.049

**Published:** 2025-04-01

**Authors:** Mikołaj Dziurzyński, Maksymilian E. Nowak, Maria Furman, Alicja Okrasińska, Julia Pawłowska, Marco Fondi

**Affiliations:** a Department of Biology, University of Florence, Via Madonna del Piano 6, Sesto Fiorentino, FI 50019, Italy; b Institute of Evolutionary Biology, Faculty of Biology, Biological and Chemical Research Centre, University of Warsaw, Żwirki i Wigury 101, Warsaw 02-089, Poland

**Keywords:** Metabolic modelling, Lipid synthesis, Biotechnology, Filamentous fungi, Fatty acids

## Abstract

Oleaginous fungi—known for their high lipid content of up to 80 % of dry mass—are of significant interest for biotechnological applications, particularly in biofuel and fatty acid production. Among these, the genus *Umbelopsis*, a common soil saprotroph of the Mucoromycota phylum, stands out for its rapid growth, low nutritional requirements, and ability to produce substantial amounts of lipids, especially polyunsaturated fatty acids (PUFAs). Despite previous studies on lipid production in *Umbelopsis*, metabolic engineering has been underexplored. This study fills that gap by presenting the first comprehensive metabolic model for *Umbelopsis* sp. WA50703, encompassing 2418 metabolites, 2215 reactions, and 1627 genes (iUmbe1). The model demonstrated a strong predictive accuracy correctly predicting metabolic capabilities in 81.05 % of cases when evaluated against experimental data. The Flux Scanning based on Enforced Objective Flux (FSEOF) algorithm was utilized to identify gene targets for enhancing lipid production. This analysis revealed 33 genes associated with 23 metabolic reactions relevant to lipid biosynthesis. Notably, the reactions catalysed by acetyl-CoA carboxylase and carbonic anhydrase emerged as prime candidates for up-regulation. These findings provide clear guidelines for future metabolic engineering efforts to optimize PUFA production in *Umbelopsis* strains.

## Highlights

• First comprehensive metabolic model of *Umbelopsis* species.
• Model includes 1627 genes, 2215 reactions, and 2418 metabolites.
• 81.05 % predictive accuracy against qualitative, experimental growth data.
• Identified 33 genes in 23 reactions to optimize PUFA in *Umbelopsis* sp. WA50703.
• Model guides PUFA and lipid production optimization for biotechnology.

## Graphical Abstract

**Figure:**
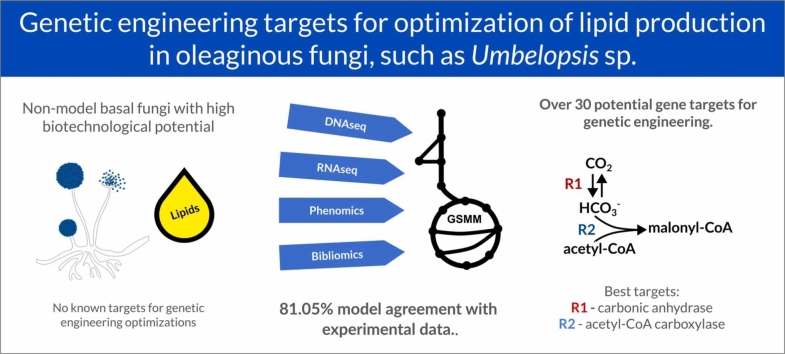

## 1 Introduction

Oleaginous fungi are notable for their high lipid content which can constitute up to 80 % of their dry mass [1]. They belong to different taxonomic groups and can be both filamentous fungi and yeasts. In natural habitats, the accumulated lipids serve primarily as the main energy storage material; they also play critical roles in processes such as symbiosis stabilization [2], [3]. The ability of oleaginous fungi to accumulate large amounts of lipids has led to extensive studies on their potential for biotechnological applications, particularly in the production of biofuels and valuable fatty acids [4], [5].

Members of the *Umbelopsis* genus are globally distributed common soil saprotrophs belonging to the Mucoromycota phylum and predominantly associated with forest soils [6], [7]. Known for their relatively rapid growth rate and low nutritional requirements, *Umbelopsis* members have garnered significant interest in biotechnological studies investigating lipid production, with a focus on polyunsaturated fatty acids (PUFAs) [8]. Although lipid production in some *Umbelopsis* strains has been thoroughly studied, the phylogeny of the genus is still unresolved, leading to several misnaming and classification biases [9]. The ability of *Umbelopsis* fungi to produce high lipid content - especially when cultured with glucose - may reach up to 74 % of dry cell weight [10]. Valuable PUFAs such as linoleic acid (Δ9,12 C18:2) and gamma-linolenic acid (Δ6,9,12 C18:3) were shown to constitute up to 18.8 % and 5.1 % of the lipid mass, respectively [11], [12].

While lipid production optimization efforts for members of the *Umbelopsis* genus have primarily focused on growth media and general culture parameters, metabolic engineering approaches have been notably absent. Unlike other oleaginous fungi which have been extensively analysed using bioinformatics to identify potential gene optimization targets [5], [13], [14], *Umbelopsis* sp. has not undergone such detailed investigations.

In depth genomic engineering analyses from other oleaginous fungi have highlighted long-chain fatty acid desaturases, elongases, and NADPH-generating reactions like the malic enzyme reaction as key targets for optimization [5], [13], [15]. Although similar potential interventions have been suggested for *Umbelopsis* [8], they have not been thoroughly explored, partially due to the limited availability of comprehensive genomic and experimental data necessary to guide such efforts. However, this gap could be addressed by reconstructing and analysing the organism's metabolic model, thus providing a valuable framework for identifying and exploring metabolic engineering targets for the future.

Metabolic models have a wide range of applications: from optimizing product synthesis, discovering novel reactions and metabolic pathways, analysing the evolution of metabolic networks, to describing the robustness of metabolic systems [16], [17], [18]. In the fungal kingdom, the models have been extensively used to understand cellular adaptation to a wide array of environmental and genetic perturbations, optimize production of various compounds, identify novel transporter proteins, and even study the metabolic determinants of the Crabtree effect [19], [20], [21], [22].

The research herein presents the first comprehensive reconstruction of the metabolic model of *Umbelopsis* sp. WA50703. The model has been rigorously validated against available experimental data and examined for potential gene modifications to enhance the production of polyunsaturated fatty acids (PUFAs). Using the Flux Scanning based on Enforced Objective Flux (FSEOF) algorithm, multiple optimization strategies were identified, highlighting two critical reactions associated with the initiation and elongation of fatty acid synthesis as primary targets for metabolic engineering.

## 2 Materials and methods

### 2.1 Genome assembly and annotation

Raw genome sequencing data of *Umbelopsis* sp. WA50703 were downloaded from the GenBank database (accession id: SRR26874108) and the reads were subjected to quality control and trimming using fastp v0.22.0 [23]. Next, the reads were assembled using SPAdes v3.15.5 assembler using the *isolate* flag [24]. The assembly’s quality was assessed using QUAST v5.2.0 with the *fungus* flag [25]. Additionally, BUSCO v5.4.2 (run with mucoromycota_odb10) and CheckM2 were used to check assembly completeness and putative assembly contamination [26], [27]. The taxonomy of the strain was confirmed by aligning ITS sequences extracted from the assembly using ITSx v. 1.1.3 versus the UNITE database [28], [29].

Structural and functional annotation was conducted using the funannotate pipeline v. v1.8.14 [30]. Briefly, repeat-masking was performed utilizing TANTAN software [31]. Masked contigs were subjected to gene calling, which was conducted using multiple programs: Augustus, GeneMark, GlimmerHMM and SNAP supplied with RNAseq evidence (European Nucleotide Archive run ID: SRR13866027) to improve prediction accuracy [32], [33], [34], [35]. Results obtained from all programs were combined and integrated using Evidence Modeler v. 1.1.1 [36]. tRNAs were predicted using tRNAscan-SE v. 2.0.9 [37]. Predicted coding sequences were annotated using the “annotate” script included in the funannotate pipeline which annotates coding sequences using multiple reference databases such as Pfam, UniProt, CAZy, MEROPS, and BUSCO groups [38], [39], [40], [41]. Additionally, the pipeline was supplied with InterProScan and EGGnogMapper results which were then integrated into the annotation [42], [43]. In the following step, the annotations of protein cellular localization based on SignalP results and of biosynthetic gene clusters based on antiSMASH results were performed [44], [45]. Finally, the annotation was manually curated using the MAISEN web application [46].

### 2.2 Draft model reconstruction and manual curation

The draft model was *de novo* reconstructed using Pathway Tools v. 26.5 a software employing a multi-stage process to identify metabolic reactions through genome annotation word-matching and protein sequence alignment with the MetaCyc database; the programme also performs a basic gap-filling process to complete metabolic pathways by adding potentially present but previously unidentified reactions [47]. The initial draft reconstruction was then manually curated following the procedure outlined by Thiele and Palsson [48]. This curation process included evaluating the feasibility of biomass precursor production, aligning the model with SBML standards, verifying reaction directionality, incorporating the mitochondrion compartment, and correcting erroneous CO₂ assimilation and energy generation reactions.

The biomass reaction was defined using a spreadsheet developed by Ye et al. in their work on the *M. alpina* model [13]. Macromolecular formulations were supplemented with data from the available literature. DNA, RNA, and amino acid contributions were calculated using the BOFdat algorithm, while lipid and carbohydrate fractions were adjusted based on experimental data from multiple studies [49].

The quality of the model was evaluated using the Memote test suite, and a FROG report was generated to ensure the reproducibility and reliability of the simulations (Supplementary Files A and B) [50].

### 2.3 Experimental data collection

The available literature was thoroughly reviewed for experimental data pertaining to growth of *Umbelopsis* sp. in various conditions. Special attention was paid to culturing experiments measuring fungal growth coupled with depletion of carbon substrates and other research testing the utilization of different carbon sources by *Umbelopsis* members.

In the first case, five articles with a total of seven different experimental datasets have been identified. Available growth and carbon source depletion curves were extracted and subjected to further calculations. Growth curves were standardized using the growthcurver v. 0.3.1 package run in RStudio v. 4.2.1 environment [51]. Maximal growth rates and carbon source uptake rates were calculated following Fondi et al. [52].

Carbon assimilation data was extracted from the work of Pawłowska et al. [53]. The data included Biolog phenotypic array results run with *U. isabellina* CBS167.80. The data covered tests conducted with 95 different carbon sources with three replicates each. Due to high data variability, positive assimilation capability was assumed only when growth was observed in at least two out of three replicates.

### 2.4 *In silico* optimization of lipids production

The Flux Scanning based on Enforced Objective Flux (FSEOF) algorithm was applied to identify metabolic reactions whose flux increases as linoleate production is enforced [54]. This approach is widely used in metabolic engineering to pinpoint bottlenecks and gene amplification targets that may enhance the biosynthesis of a desired compound. To ensure robustness, FSEOF analysis was conducted on 59 different metabolic model versions, each supplied with a distinct carbon source. The simulations were performed using the RAVEN Toolbox 2.8.4 in MATLAB R2023 [55], [56]. The analysis systematically enforced a shift from biomass production (Biomass_reaction_1) toward linoleate secretion (DM_linoleate_c), allowing the identification of reactions that exhibited a consistent increase in flux. To filter the most relevant targets, we retained reactions that appeared in at least five independent analyses and had an FSEOF slope value greater than 2, ensuring a focus on the most significant pathways contributing to linoleate production.

### 2.5 Data availability

The iUmbe1 and iUmbe1-slim models, along with custom tests are available under the following URL: https://github.com/mdziurzynski/iUmbe1-GEM. Genome assembly and annotation are available in the NIH NCBI GenBank database under the following accession number: GCA_964291815.

## 3 Results

### 3.1 Genome assembly and annotation results

Quality assessment of the raw sequencing data indicated high quality, with over 99 % of reads passing all filtering steps. The assembled genome of *Umbelopsis* sp. WA50703 comprised 234 contigs with a mean sequencing depth of 134x and an average GC content of 41.95 %. The L50 and N50 metrics were 10 and 750,386, respectively (Supplementary Table A). BUSCO analysis revealed 95 % genome completeness, with 2.3 % of genes fragmented and 2.7 % missing. CheckM2 showed genome completion at 89.43 % with 8.63 % of possible contamination. Species level identification enabled the classification of the strain within the *U. isabellina* section, however, as the strain does not form a monophyletic group with *U. isabellina* type material, therefore, we refrain from using any species name for WA50703 strain.

**Fig. 1:**
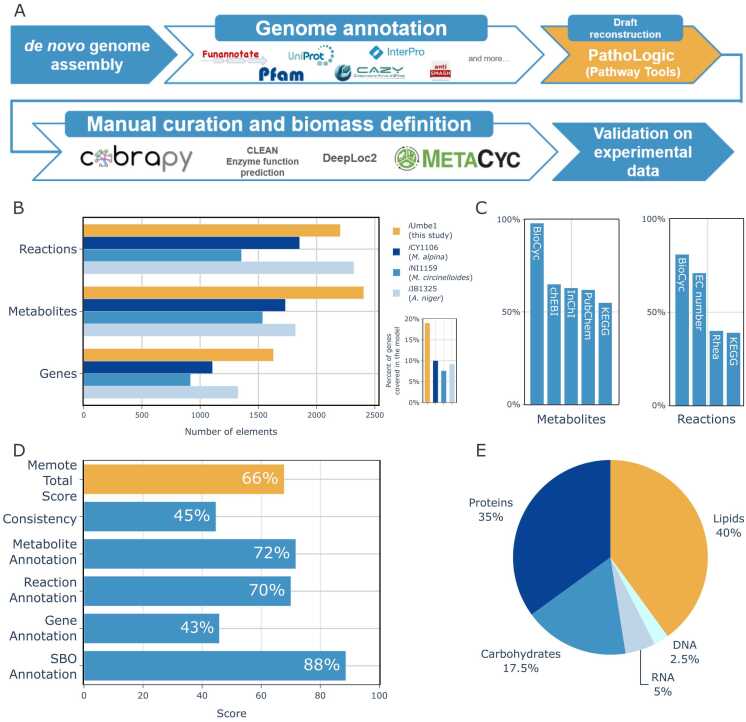
Model reconstruction pipeline and model metrics. A: main steps of the model reconstruction pipeline, B: comparison of iUmbe1 reconstructed in herein with three other high-quality fungal models; C: iUmbe1 reactions and metabolites annotation coverage; D: Memote results for iUmbe1; E: main biomass precursors and their contributions.

During genome annotation, 8693 genes were identified, including 8548 encoding mRNAs and 145 encoding tRNAs. A total of 7271 genes were annotated with at least one functional domain, and detailed protein functions were assigned to 2832 of them. Additionally, 1464 proteins were annotated with an EC number. Overall, only 1277 proteins remained without any functional annotation. The detailed annotation results are available in Supplementary Table A.

### 3.2 Model description

The reconstructed model was named iUmbe1 and consisted of 2215 reactions, 2418 metabolites and covered 1627 genes (Fig. 1). The model is highly annotated as over 75 % of all reactions and nearly 100 % of metabolites were additionally annotated with an appropriate BioCyc identifier. Beside BioCyc, metabolites and reactions were also annotated with identifiers to the following classifications and databases: Enzyme Commission number, Chemical Entities of Biological Interest (chEBI), International Chemical Identifier (InChl), PubChem, KEGG, Rhea, Chemical Abstracts Service number (CAS) and others.

The iUmbe1 model consists of three major compartments: extracellular space, cytosol and mitochondrion. Using DeepLoc 2.0, a deep learning algorithm for predicting subcellular localizations of eukaryotic proteins, it was possible to assign reactions and by extension the metabolites into their corresponding compartments. In total 67, 1591 and 389 reactions were localized and sorted in the extracellular compartment, cytosol and mitochondrion, respectively. Additionally, there were 168 reactions related to transport which were examined manually. Only relevant and feasible reactions selected according to the MetaCyc or the Transporter Classification Database were added.

The biomass equation was curated using experimental data available in the literature from studies on *Umbelopsis* strains. Breakdown of biomass precursors is presented in Table 1. Although exact data for DNA, RNA, and protein content were 155 unavailable, their biomass contributions were estimated based on genome content, using a methodology similar to that employed by the BOFdat Python package [49]. Data published by Muszewska et al. (2021) provided a detailed estimation of the carbohydrate fraction predominantly composed of glucose, galactose, mannose, fucose, and N-acetylglucosamine [7]. Growth associated maintenance energy, calculated as described by Thiele and Palsson, was set to 153 mmol ATP/g_DW_ [48]_._

**Table 1:** Biomass precursors and their constituent components. Percentages in brackets represent the contribution of biomass precursors to total biomass and the contribution of individual components to their respective biomass precursors.

**Biomass precursors**	**Biomass precursors components**
**DNA (2.5 %)**	dATP (28.6 %)
	dCTP (22.4 %)
	dGTP (19.6 %)
	dTTP (29.4 %)
**RNA (5 %)**	ATP (26.4 %)
	CTP (25.2 %)
	GTP (20.1 %)
	UTP (28.2 %)
**Protein (35 %)**	Alanine (7.6 %)
	Arginine (5.2 %)
	Asparagine (4.7 %)
	Aspartic acid (6.0 %)
	Cysteine (1.2)
	Glutamate (6.4 %)
	Glutamine (4.6 %)
	Glycine (5.7 %)
	Histidine (2.4 %)
	Isoleucine (5.7 %)
	Leucine (8.8 %)
	Lysine (5.89 %)
	Methionine (2.4 %)
	Phenylalanine (3.7 %)
	Proline (5.1 %)
	Serine (8.3 %)
	Threonine (5.9 %)
	Tryptophan (1.2 %)
	Tyrosine (3.2 %)
	Valine (6.1 %)
**Lipids (40 %)**	Triglycerides (84.6 %)
	Diglycerides (4.8 %)
	Free fatty acids (4.8 %)
	Sterols (1.0 %)
	Phosphatidylethanolamines (0.7 %)
	Phosphatidylcholines (2.8 %)
	Phosphatidylinositols (0.7 %)
	Phosphatidylserines (0.6 %)
	Phosphatidic acid (0.2 %)
**Carbohydrates (17.5 %)**	Fucose (23.7 %)
	Glucosamine (42.9 %)
	Galactose (4.1 %)
	Glucose (26.8 %)
	Mannose (2.5 %)

Since most experimental data regarding *Umbelopsis* focus on the organism's lipid production under various medium and environmental conditions, the lipid fraction was curated with the highest accuracy and detail [8], [9], [57]. The data enabled a comprehensive partitioning of the lipid fraction into neutral lipids and phospholipids, along with detailed contributions from seven different fatty acids and five classes of phospholipids. However, as the lipid data were derived from highly optimized cultures, the exact contributions of each compound were adjusted for a minimal medium environment. A full description of biomass fractions and their contributions is available in Supplementary Table B.

To enhance computational efficiency, a streamlined version of the model —iUmbe1-slim—was constructed by removing all orphan metabolites and the reactions associated with them. This curation, however, came at the significant cost of discarding numerous reaction–gene associations that reflect the organism’s broader metabolic capabilities. The lean model is recommended for use in computationally intensive analyses where performance is prioritized over completeness.

The reconstructed models were evaluated using Memote, a comprehensive test suite for genome-scale metabolic models, and received an overall score of 66 % [50]. The highest scores were achieved in data annotation categories, including "Metabolite Annotation" and "Reaction Annotation," while the lowest score was in "Consistency". A detailed comparison of the scores obtained in the "Consistency" section of the Memote test suite, illustrating how the model performs relative to different GSMMs of fungi, is provided in Supplementary Figure 1. The complete Memote report is available as Supplementary File A. Additionally, standard-GEM template was used to set up a GitHub repository to host the latest version of the model. The models can be accessed at the following link: https://github.com/mdziurzynski/iUmbe1-GEM.

### 3.3 Model validation on experimental data

**Fig. 2:**
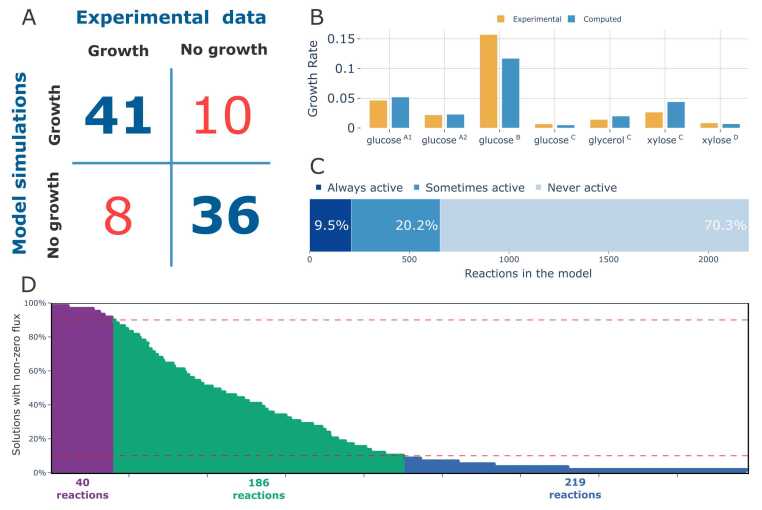
Model validation results. A: comparison of Biolog phenotypic microarray results for *U. isabellina* CBS167.80 with iUmbe1 model simulations; B: comparison of experimental and computed growth rates of *U. isabellina* with different carbon sources, ^A1^- flask cultures data from Chatzifragkou A. et al# [10], ^A2^- cultures held in a 3 litre bioreactor from Chatzifragkou A. et al# [10], ^B^- cultures held in a 3 litre bioreactor from Meeuwse et al# [58], ^C^- flask cultures data from Fakas S. et al# [59], ^D^- flask cultures data from Gardeli C. et al# [11]; C: utilization of model reactions observed in simulations conducted with different carbon sources, D: activity of “Active at least once” reactions set among all 59 simulations with different carbon sources. Red dashed lines separate the 10 % and 90 % activity thresholds, dividing the graph into three groups of near core reactions (violet), carbon source type related reactions (green), carbon-source specific reactions (blue).

The reconstructed model was evaluated against available experimental carbon assimilation data extracted from the work of Pawłowska et al. [53] (Fig. 2A). The data contained information about the *U. isabellina* CBS167.80 growth on 95 carbon sources, i.e. 7 amines/amides, 13 amino acids, 15 carboxylic acids, 46 carbohydrates, 5 polymers, and 9 other compounds. All simulations were conducted with four exchange reactions, reflecting essential compounds present in a minimal medium for *U. isabellina*, left unconstrained. The exchange reactions concerned phosphate, sulphite, sulphate and ammonium. Additionally, exchanges for carbon dioxide, oxygen, water and hydrogen ions were also set open.

The analysis demonstrated an overall agreement of 81.05 % between the experimental results and a simulations. The model accurately predicted the growth on 41 carbon sources that was also proven *in vitro*; furthermore, it correctly predicted no growth on 36 carbon sources, also aligning with the *in vitro* results. However, discrepancies for 18 carbon sources were noted: 8 displayed no growth *in silico*, while 10 showed growth *in silico*, contrary to the experimental data.

The former group of the 8 discrepant carbon sources included D-glucuronic acid, Tween 80, α-cyclodextrin, β-cyclodextrin, dextrin, β-methyl-D-glucoside, L-rhamnose, and sebacic acid. For these compounds, no transporters were detected suggesting either transportation via unknown mechanisms or proteins, or initial extracellular degradation by unidentified secreted enzymes. Specifically, no substrate-specific enzyme responsible for the degradation of D-glucuronic acid was identified. Tween 80 likely undergoes non-specific degradation by esterases, lipases, and cutinases. The absence of cyclodextrinase (EC 3.2.1.54) in the genome assembly precluded the inclusion of cyclodextrin (both α- and β-cyclodextrin) degradation reactions in the model. Additionally, no relevant enzymes for the degradation pathways of L-rhamnose or β-methyl-D-glucoside were identified. Enzymes required for the degradation of alpha, omega-dicarboxylate acids (sebacic acid) such as EC 6.2.1.23 and EC 6.2.1.10 were also absent.

The latter group of carbon sources that were predicted *in silico* as able to sustain *Umbelopsis* growth consisted of xylitol, fumaric acid, β-hydroxy-butyric acid, α-keto-glutaric acid, L-lactic acid, succinic acid, L-aspartic acid, L-proline, L-serine, and L-threonine. Most of those compounds participate in core metabolic pathways and therefore their positive and, simultaneously, incorrect assimilation result most likely arises from the misidentification of their respective transporter proteins. The full table comparing *in silico* simulations with experimental data results is available as Supplementary Table C.

Biolog experimental results were also used to evaluate the overall utilization of reactions included in the model. Information about active and inactive reactions from feasible *in silico* solutions for 59 different carbon sources were pooled and analysed (Fig. 2A). The results showed that, on average, each solution contained 336 active reactions. Analysis of the pooled data analysis revealed that in all 59 different solutions 212 reactions were always active (9.5 %), 450 reactions were active at least once (20.2 %) and 1553 reactions were never active (70.3 %) (Fig. 2C). While the “active at least once” reactions were heavily associated with the supplied carbon source with a clear division between carbohydrates and amino acids further analysis showed interesting recurrent patterns. Out of the 450 auxiliary reactions (the “active at least once” set), 41 (9.11 %) were extremely close in their activity patterns to the core reactions, as they were inactive in at most 10 % of the investigated solutions (Fig. 2D). The following 188 reactions (41.78 %) could be interpreted as necessary for the assimilation of different carbon source types such as amino acids or carbohydrates. Their activity wams observed in no less than 10 % and no more than 90 % of solutions. Finally, the remaining 221 reactions, which constituted 49.11 % of the auxiliary set, were interpreted as rarely active but vital for the metabolism of specific carbon sources, as they were active in no more than 10 % of solutions. A detailed pathway analysis of this latter group identified three pathways that were active exclusively when growth was simulated on very specific carbon sources: adenosine degradation through urate and allantoine (10 rare reactions, BioCyc IDs: SALVADEHYPOX-PWY, PWY-5691, PWY-5694), D-galacturonate degradation (5 rare reactions, BioCyc ID: PWY-6491), and L-arabinose degradation (3 reactions, BioCyc ID: PWY-5515).

Furthermore, we evaluated the capability of the reconstruction to quantitatively resemble the growth rates of *Umbelopsis sp*. Accordingly, growth rates predicted by the model were compared against growth rates extracted from growth curves available in the literature. Biomass equation across all calculations remained the same, as described in Section 3.2. Although the experimental data came from studies conducted with different culture conditions and even different *U. isabellina* strains, the results showed high similarity between *in silico* and *in vitro* growth rates. Experimental data covered four experiments established with glucose as the main carbon source, two with xylose and one with glycerol. Fig. 2B presents growth rates from each experiment and their computed pair from the model simulations. Overall, the model showed remarkable accuracy in predicting the growth rates of *U. isabellina* on known carbon sources. Only in two out of seven comparisons the difference between predictions exceeded 30 %, as in the cases of glucose^B^ and xylose^D^ experiments the experimental growth rate was 1.3 higher and 0.6 lower, respectively. In the remaining cases, the simulation results and experimental values resembled one another (Fig. 2B). Overall, the aforementioned results suggest that the iUmbe1 reconstruction can reliably predict *U. isabellina* growth phenotypes both qualitatively and quantitatively.

### 3.4 Lipid production optimization – case study

**Fig. 3:**
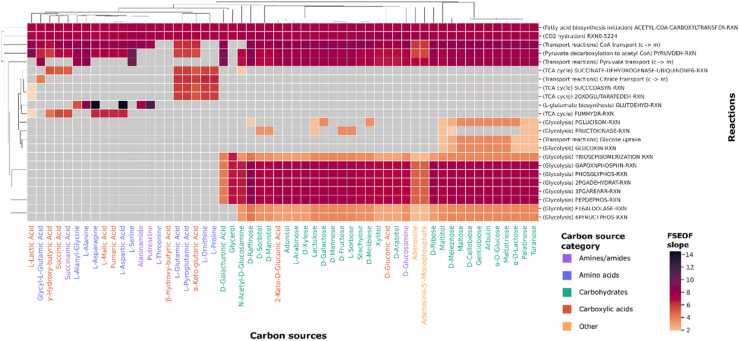
Heatmap depicting FSEOF analysis results. Slope values indicate the level of co-upregulation of a given reaction with an increase in the production of linoleate. Grey tiles indicate that a given reaction was not identified as a potential target for improvement for a given carbon source. Other than transport reactions, the names are MetaCyc reaction identifiers.

To identify metabolic engineering targets for improving lipid biosynthesis, we applied the Flux Scanning based on Enforced Objective Flux (FSEOF) algorithm [54]. FSEOF is a constraint-based analysis method designed to identify metabolic reactions whose flux increases as the production of a target metabolite is enforced. By systematically shifting the model’s objective from biomass production to linoleate biosynthesis, we aimed to pinpoint reactions that play a crucial role in lipid metabolism across different carbon sources. This approach is particularly useful in oleaginous fungi, where lipid accumulation is tightly linked to central metabolism. The analysis was conducted on 59 metabolic model variants, each representing different carbon substrates, to ensure robustness and applicability of the findings. FSEOF analysis returned 297 potentially interesting gene targets, most of which were reported for only one specific carbon source. To identify the most important and the most prevalent gene targets, a final reaction set was filtered to only contain reactions that were reported for at least 5 different carbon sources. Additionally, a FSEOF slope threshold of greater than 2 was applied to ensure that only the reactions with the highest impact were retained. Fig. 3 presents a heatmap describing this set of reactions and includes 23 reaction instances retrieved after simulations with 56 different carbon sources. There were 5 reactions that showed up in nearly all solutions as potential targets for improvement.

The next six reactions were associated with the TCA cycle and introduction of L-glutamate into the TCA cycle. These reactions were reported mainly in solutions obtained for growth simulations with amino acids and amides/amines as the main carbon sources. The last and, at the same time, the largest set comprised of 12 reactions, 11 of which constituted the core of glycolysis. As expected, those reactions were reported mostly in growth solutions set with carbohydrates as their main carbon sources.

The 23 reactions highlighted by FSEOF within the set are encoded by 33 different genes, some of which encode multi-protein complexes (Supplementary Table D). Three of the reactions lacked gene annotation and they were “Pyruvate transport (c -> m)”, “Citrate transport (c -> m)” and “FRUCTOKINASE-RXN”.

## 4 Discussion

The high biotechnological potential of different *Umbelopsis* strains has been continuously investigated for over 20 years; however, the primary focus of these analyses was the optimization of exogenous factors - such as culture conditions - for the production of various value-added compounds, predominantly PUFAs [8]. Although the organism exhibits exceptional oleaginicity and grows well on different low-cost carbon sources, further and more detailed optimization projects are substantially hampered by the limited availability of a suitable genetic toolkit and *in silico* insights that could guide such efforts.

In this study we present the first *in silico* metabolic model of *Umbelopsis* sp. WA50703. High attention was paid to both genome and model annotation in this reconstruction, in order to increase the interoperability and utility of the model. The genome assembly analysis confirmed the expected genome size of 22.74 Gbp, comparable with other *Umbelopsis* genome assemblies available in the GenBank database. Although BUSCO analysis yielded a high, 95 % genome completeness result, CheckM2 contamination analysis showed 8.63 % contamination. Further analysis using BlobTools2 proved that the contamination was detected most likely due to the incompleteness and incorrectly annotated fungal sequences in the reference databases as fungal genomes sequencing lags significantly behind other organism groups [60].

Detailed genome annotation resulted in exceptionally high genome coverage in the iUmbe1 model (20 % of all genes were present in the model) when compared with other high-quality fungal models (7–10 % of genes) [13], [61]. Although the model contains a similar number of metabolites and reactions compared to others, it has been reconstructed with only three compartments: extracellular, cytosolic, and mitochondrial. Eukaryotic models typically include additional compartments, such as the periplasmic space, peroxisome, nucleus, or Golgi apparatus. However, in the case of iUmbe1, this further division of reaction space was omitted due to the lack of experimental data needed to accurately assign reactions to these compartments. Without data on inter-compartmental transport capabilities, incorporating additional compartments would result in artificial and unreliable pathway structures and flux flows that may not accurately reflect biological reality. It is important to note that, should there be a reason and sufficient data to add new compartments in the future, the model's reaction and metabolite annotations are designed to facilitate this task.

While the experimental validation results showed a high degree of accordance with model simulations, they also highlighted significant areas for improvement. The experimental data collected from publicly available resources for *Umbelopsis* species proved invaluable for model curation and testing. However, to eliminate inconsistencies between experimental results and simulations, future experiments must be conducted in a more strain-specific manner as it was done with *Aspergillus niger* by Brandl J. et al. [61]. Variability in growth rates obtained from glucose growth experiments can be attributed to substantial differences in experimental setups.

For example, the experiment with the highest growth rate had several differing conditions: a much higher initial concentration of nitrogen source (20 mM vs. 4 mM), a pre-culturing step to adapt the fungus to culture conditions, active aeration, larger volumes (2000 ml vs. 50 ml), and used a different *U. isabellina* strain (CBS 194.28 vs. ATHUM 2935) [58], [59]. The growth rate reported by Meeuwse et al. was significantly higher (μ= 0.157) than the values predicted by *in silico* simulations (μ=0.117) [58]. This finding is intriguing because genome-scale metabolic models (GEMs) are designed to estimate the maximum theoretical growth rate—representing the upper limit of an organism’s metabolic potential—while experimental cultivation conditions typically yield much lower growth rates. The observed discrepancy likely arises from differences between the strain used to construct the GEM and the strain employed in the experimental study. The genus *Umbelopsis* is renowned for its metabolic diversity; even among well-studied species like *Umbelopsis isabellina*, *U. ramanniana*, and *U. vinacea*, significant variation exists in metabolic capabilities, optimal growth temperatures, and nutrient preferences. [9].

Additionally, it is important to note that *Umbelopsis* has only recently been recognized for its facultative yet persistent endohyphal bacteria which can significantly expand its metabolic capabilities [62]. Therefore, before generating experimental data, it should be confirmed that the strain in use is free of these symbionts.

The model’s validation confirmed its readiness for guiding metabolic engineering strategies, and FSEOF analysis proved to be a valuable tool for identifying potential gene amplification targets. By systematically shifting the model’s objective function from biomass production to linoleate biosynthesis, FSEOF identified 23 reactions that were strongly correlated with increased lipid production. These reactions spanned three major metabolic pathways: glycolysis (especially for carbohydrate-based media), the TCA cycle (for amino acid-based media), and fatty acid biosynthesis. Among them, ACETYL-COA-CARBOXYLTRANSFER-RXN, RXN0–5224 and PYRUVDEH-RXN emerged as key targets, appearing consistently across nearly all tested conditions. Acetyl-CoA carboxylase (ACC) catalyses the ATP-dependent carboxylation of acetyl-CoA to malonyl-CoA, the first committed step in fatty acid biosynthesis. Since malonyl-CoA is the direct precursor for lipid synthesis, increasing ACC activity has been widely recognized as a key strategy to enhance lipid accumulation in oleaginous fungi and other lipid-producing organisms. Previous studies have shown that ACC overexpression significantly increases lipid yields in fungi, bacteria, and algae, with reported improvements ranging from 1.16-fold in *Chlamydomonas reinhardtii* to over 6-fold in *Escherichia coli* [63], [64], [65], [66], [67].

Carbonic anhydrase (CA), on the other hand, is a metalloenzyme responsible for the reversible conversion of CO₂ to bicarbonate, which is a required substrate for ACC. By increasing bicarbonate availability, CA indirectly supports malonyl-CoA biosynthesis and enhances overall carbon flux toward fatty acid production. Studies on fungal and algal lipid production have demonstrated that overexpressing CA can improve lipid synthesis by boosting bicarbonate availability and facilitating ACC activity [64], [68], [69].

The last reaction was mitochondrial PYRUVDEH-RXN which is catalysed by pyruvate dehydrogenase complex (PDC). This reaction combines pyruvate with coenzyme A into acetyl-CoA with the release of NADH. While it plays a major role in feeding carbon to the TCA cycle in the mitochondrion, metabolic engineering efforts focused on repurposing it as a part of cytosolic, multistep, synthetic pathway. This engineered pathway converts pyruvate to acetyl-CoA while simultaneously producing NADPH — a critical cofactor for fatty acid chain elongation — to boost lipid biosynthesis [70].

All three of those reactions were identified across nearly all tested growth conditions, suggesting that their upregulation would enhance lipid production independent of the carbon source, making them robust targets for metabolic engineering. These findings align with previously reported metabolic engineering efforts in oleaginous microbes and provide a strong basis for future strain optimization of *Umbelopsis* WA50703 for the production of lipids, and especially PUFAs.

## 5 Conclusions

In this work the metabolic model of oleaginous fungi *Umbelopsis* sp. WA50703 was reconstructed with special attention paid to metabolites, reactions and gene annotation quality. Experimental validation of the model showed 81.05 % concordance with phenotypic microarray data and 66 % Memote Total Score. A case study analysis using the model, aiming at identification of possible targets for improvement to increase PUFAs synthesis yielded 23 reactions, out of which three have been chosen as the most promising as they were identified on nearly all tested carbon sources. The model not only provides a foundation for metabolic engineering of *Umbelopsis* species but also offers a practical tool for optimizing microbial lipid biosynthesis, with potential applications in biofuel production and the sustainable synthesis of high-value fatty acids for industrial use.

## Funding

M.D. was funded by European Molecular Biology Organization Postdoctoral Fellowship (grant no. ALTF 565–2021).

## CRediT authorship contribution statement

**Mikołaj Dziurzyński:** Conceptualization, Methodology, Investigation, Formal analysis, Writing - Original Draft, Visualization, Project administration. **Maksymilian E. Nowak:** Data curation, Investigation. **Maria Furman:** Investigation, Methodology. **Alicja Okrasińska:** Methodology, Writing - Review & Editing. **Julia Pawłowska:** Conceptualization, Writing - Review & Editing, Resources. **Marco Fondi:** Conceptualization, Methodology, Writing - Review & Editing, Resources.

## Declaration of Generative AI and AI-assisted technologies in the writing process

During the preparation of this work the authors used ChatGPT 4o in order to enhance language quality, grammar, and style. After using this tool/service, the authors reviewed and edited the content as needed and take full responsibility for the content of the publication
