# Supplementary figures and images for: Insights into optimization of oleaginous fungi – genome-scale metabolic reconstruction and analysis of Umbelopsis sp. WA50703
Source: Comput Struct Biotechnol J. 2025 Apr 1;27:1431–9. doi: 10.1016/j.csbj.2025.03.049 (PMC12002602; doi:10.1016/j.csbj.2025.03.049)

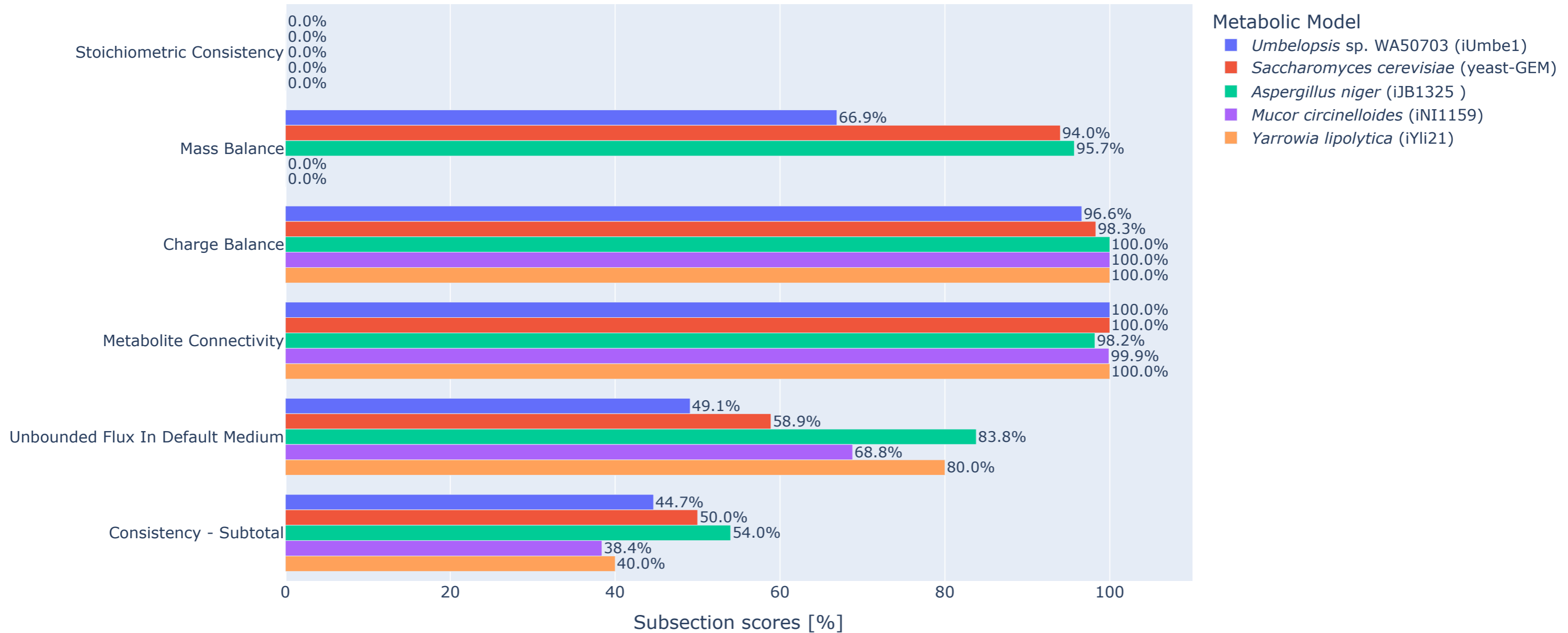

Supplement: Supplementary file 1 — Supplementary material [file mmc1.pdf]
